# A New and Quick Furniture Polish

**Published:** 1894-04

**Authors:** 


A NEW AND QUICK FURNITURE POLISH.—In the German patent list we find the following specification for a patent for a new furniture polish, issued to Paul Theiol, of Copenick, near Berlin:

| | |
|---|---|
| Resin of guaiac | 125 parts. |
| Gum benzoin | 125 parts. |
| Shellac | 30 parts. |
| Linseed oil | 150 parts. |
| Benzin | 30 parts. |
| Alcohol or wood spirit | 3000 parts. |

Mix and dissolve.

The polish is applied with a sponge or brush, and the object is let stand for a half hour. A linen cloth moistened with oil is then used as a rubber, and a brilliant polish is obtained which is said to be very lasting, and is unaffected by water or other substances which usually injure varnish. Another advantage of it is that it may be applied to woods that have never been varnished or polished, and gives a result equal to the best French polish. No skill is said to be requisite in its use. The rubber must be of linen, and oiled only sufficiently to prevent its sticking when first applied.

